# Impacto de la donación de sangre del cordón umbilical sobre la hemorragia postparto y la salud neonatal

**DOI:** 10.23938/ASSN.1125

**Published:** 2025-10-23

**Authors:** Rafael Esteban-Bañó, Raimunda Montejano-Lozoya, Carlos Saus-Ortega, Pedro García-Martínez, Pilar Solves Alcaina, Alfredo Perales-Marín

**Affiliations:** 1 Escuela de Enfermería La Fe Universidad de Valencia Valencia España; 2 Grupo de Investigación Arte y Ciencia del Cuidado (GREIACC) Valencia España; 3 Instituto de Investigación Sanitaria La Fe (IIS La Fe) Valencia España; 4 Hospital Universitario y Politécnico de La Fe Servicio de Hematología Valencia España; 5 CIBERONC Instituto de Salud Carlos III Madrid España; 6 Universidad de Valencia Facultad de Medicina Departamento de Pediatría, Obstetricia y Ginecología Valencia España; 7 Hospital Universitario y Politécnico de La Fe Servicio de Obstetricia y Ginecología Valencia España

**Keywords:** Sangre de Cordón Umbilical, Donación de Sangre, Hemorragia postparto, Recién Nacido, Puntaje de Apgar, Fetal Blood, Blood Donation, Postpartum Haemorrhage, Infant, Newborn, Apgar Score

## Abstract

**Fundamento:**

La donación de sangre de cordón umbilical ha incrementado globalmente, siendo fundamental garantizar la seguridad materna y neonatal. Este estudio evalúa su relación con hemorragia postparto, transfusiones, duración del alumbramiento, retención placentaria y estado neonatal.

**Material y métodos:**

Estudio de cohortes prospectivo realizado en el Hospital Universitario y Politécnico La Fe (Valencia, España) entre 2009 y 2015. Se incluyeron gestantes adultas de ≥37 semanas, bajo riesgo obstétrico y parto único espontáneo; la donación fue voluntaria. Se analizaron variables maternas (incluyendo pérdida sanguínea, descenso de hemoglobina y hematocrito, transfusiones, duración del alumbramiento, retención placentaria) y neonatales (pH umbilical y test de Apgar).

**Resultados:**

Participaron 144 mujeres (73 donantes). La duración desde el parto hasta el final de la sutura (30,69±16,37 vs 22,91±12,19 minutos; p<0,001) y del alumbramiento (11,11±10,05 vs 7,99±5,92 minutos; p=0,10) fue mayor en las donantes. No hubo diferencias significativas en retención placentaria, extracción manual de la placenta, ni mayor pérdida sanguínea ni transfusiones. Las dos transfusiones en el grupo de donantes se asociaron a retención placentaria con extracción manual de la placenta y atonía uterina. No se observaron otras diferencias entre los grupos.

**Conclusiones:**

La donación de sangre del cordón umbilical no incrementa la necesidad de transfusiones sanguíneas, la duración del alumbramiento, la retención o extracción manual de la placenta, y no afecta al estado neonatal.

## Abstract (en)

**Background:**

Umbilical cord blood donations has increased worldwide, raising concerns about maternal and neonatal safety. This study aims to evaluate its impact on postpartum hemorrhage, transfusion requirements, and duration of delivery, placental retention, and neonatal outcomes.

**Materials and methods:**

A prospective observational study was conducted at *Hospital Universitario y Politécnico La Fe* (Valencia, Spain) between November 2009 and February 2015. Eligible participants were adult women with ≥37 weeks´ gestation, low obstetric risk, and single spontaneous labor. Cord blood donation was voluntary. Maternal variables analyzed included blood loss, hemoglobin and hematocrit decline, need for transfusion, length of labor, and placental retention. Neonatal variables included umbilical pH and Apgar test.

**Results:**

A total of 144 women were included (73 donors). Donors had a longer interval from delivery to the end of suturing (30.69±16.37 vs. 22.91±12.19 minutes; p<0.001), and of delivery (11.11±10.05 vs. 7.99±5.92 minutes; p=0.10), No significant differences were observed in hemodynamic variables, placental retention, or manual placental extraction. There were no signi-ficant differences in blood loss or need for transfusion, although the two transfusions in the donor group were associated with placental retention requiring manual placental extraction and uterine atony. Neonatal outcomes did not differ between groups.

**Conclusions:**

Umbilical cord blood donation does not increase maternal risk of transfusion, duration of delivery, or manual retention or removal of the placenta, and it does not adversely affect neonatal outcomes.

## INTRODUCCIÓN

La donación de la sangre del cordón umbilical (DSCU) es una técnica realizada inmediatamente después del nacimiento que consiste en extraer sangre de los vasos placentarios mediante punción de la vena umbilical^1^. Las células madre del cordón umbilical extraídas, presentan un notable potencial terapéutico a través de su trasplante alogénico, siendo efectivas en el tratamiento de una amplia variedad de enfermedades, tanto malignas como no malignas, incluyendo leucemias agudas y crónicas, linfomas, anemia aplásica, anemia falciforme, talasemia mayor y otros trastornos genéticos y adquiridos^1^. El incremento global en la cantidad de donaciones^2^ confirma la importancia de asegurar la seguridad de este procedimiento tanto para la madre como para el recién nacido. Si bien es cierto que el número de trasplantes alogénicos de sangre de cordón ha disminuido en los últimos años y que el trasplante autólogo y alogénico relacionado tienen bastantes limitaciones, la sangre de cordón es una fuente de células progenitoras de potencial uso investigativo y terapéutico en diferentes estrategias de terapia celular^3^.

Las mujeres que participan en la DSCU suelen ser gestantes con bajo riesgo obstétrico^4^. Es fundamental considerar que el postparto inmediato es un periodo crítico con alta morbimortalidad materna^5^^-^^7^, siendo la hemorragia postparto una de las complicaciones más severas. La hemorragia postparto precoz se define como una pérdida de sangre ≥500 mL o una disminución de hemoglobina de 4 g/dL o de 10 puntos en el hematocrito dentro de las 24 horas postparto^5^^,^^8^, que actualmente causa el 8% de las muertes maternas en las regiones desarrolladas y el 20% en las regiones en desarrollo^7^. Puede surgir de manera inesperada y sin factores de riesgo previos^9^, si bien su riesgo es mayor cuanto más prolongada sea la tercera fase del parto o del alumbramiento placentario^9^. Dado que la DSCU se realiza en un momento de alta susceptibilidad para la hemorragia postparto precoz, es crucial evaluar su impacto en este contexto.

Uno de los aspectos relevantes preventivos de la hemorragia postparto es la duración de la tercera fase del parto o de alumbramiento placentario, siendo mayor el riesgo de pérdida sanguínea y hemorragia postparto cuanto más prolongado sea este periodo^9^.

Se ha postulado que el volumen intraplacentario después del nacimiento puede influir en la duración del alumbramiento, y se trabaja con esta hipótesis. Determinadas prácticas disminuyen este volumen: DSCU, pinzamiento tardío del cordón, drenaje libre de la sangre del cordón, nacimiento Lotus -donde el cordón umbilical no se corta al nacer, sino que se deja unido a la placenta hasta que se seca y se desprende de manera natural^10^^)^ - y el ordeño del cordón umbilical en sentido fetal (*milking*); el volumen puede aumentar con la compresión del cordón umbilical en sentido cefálico y la inyección de suero fisiológico acompañado de oxitócicos^11^.

La DSCU está estrechamente relacionada con el momento del pinzamiento del cordón, ya que este procedimiento requiere que el cordón sea pinzado antes de la extracción de la sangre para garantizar un volumen adecuado de muestra. Durante el periodo de realización del estudio, las sociedades científicas recomendaban el pinzamiento precoz^12^, aunque ha sido una cuestión en continuo debate hasta la actualidad.

Los estudios previos han mostrado resultados variables en cuanto a la hemorragia postparto precoz, la pérdida sanguínea y la duración del alumbramiento; la mayoría han utilizado la técnica de drenaje de sangre del cordón umbilical, que implica pinzar el cordón tras el nacimiento, cortarlo y dejar drenar la sangre de la placenta a un recipiente^13^^-^^15^.

El manejo activo del alumbramiento incluye la administración de un oxitócico postparto y el control de tracción del cordón umbilical junto con una contrapresión suprapúbica, siendo el estándar de oro a nivel mundial. Sin embargo, la técnica de la DSCU puede prolongar el tiempo de alumbramiento^16^ y, potencialmente, afectar tanto a la madre como al neonato. La seguridad del recién nacido en la DSCU se ha evaluado mediante el control de variables clínicas, como el test de Apgar y la valoración de la hipoxia al nacimiento mediante la medición del pH de la sangre de cordón, siendo ambos métodos fiables^17^.

Este estudio tiene como objetivo principal evaluar la seguridad de la donación de sangre del cordón umbilical para la madre y el neonato mediante la cuantificación de la hemorragia materna precoz postparto, la necesidad de transfusiones sanguíneas, la duración del alumbramiento, la incidencia de retención placentaria y su extracción manual, así como la valoración del test de Apgar a los cinco y diez minutos tras el nacimiento.

## MATERIAL Y MÉTODOS

### Diseño y muestra

Estudio de cohortes prospectivo no aleatorizado realizado con mujeres de parto para estudiar la relación de la DSCU con la hemorragia postparto precoz y distintas variables maternas y neonatales. Se desarrolló en el Hospital Universitario Politécnico de La Fe de Valencia (España), entre noviembre de 2009 y febrero de 2015.

Se incluyeron todas las mujeres con gestación de ≥37 semanas, de bajo riesgo obstétrico según control básico del embarazo^18^, con parto único de vértice y espontáneo, y mayores de 18 años. Se excluyeron las mujeres con una historia de hemorragia postparto previa y hemorragia anteparto actual, gestación múltiple, cirugía uterina previa (cirugías extensas, malformativas o que afectaran al volumen uterino), temperatura >38ºC en el parto (tras dos lecturas), parto con anestesia general, hipertensión inducida del embarazo, muerte intrauterina, anormalidades de la hemostasia o tratamientos con anticoagulantes, peso fetal >4.000 gramos, miomas uterinos/alteraciones uterinas, hidramnios, dificultades de comunicación, malformación fetal mayor, circular de cordón umbilical apretada con sección en el parto, infecciones STORCH, y partos instrumentados. Además, también se aplicaron los criterios de exclusión propios del grupo de donación a las mujeres no donantes^19^.

Se calculó el tamaño para obtener una muestra representativa a partir de un universo de 4.726 partos. Para ello, se estableció un nivel de confianza del 95%, un error máximo admisible del 5% y una heterogeneidad del 20%, asumiendo una hipótesis conservadora superior a la descrita en la literatura^20^. El tamaño muestral calculado fue de 250 procesos de parto distribuidos en dos grupos de igual tamaño.

El estudio fue aprobado por el comité ético del Hospital Universitario y Politécnico de La Fe de Valencia (dictamen nº 0272). Todos los datos recogidos fueron anonimizados, almacenados en una base de datos para su posterior análisis estadístico y custodiados por el investigador principal, garantizando la confidencialidad de los datos, según la Ley 41/2002, de 14 de noviembre, básica Reguladora de la autonomía del paciente y de derechos y obligaciones en materia de información y documentación clínica. La participación fue voluntaria previa firma del consentimiento informado^21^.

### Procedimiento y variables maternas

Se realizó una historia médica y obstétrica detallada de la madre, incluyendo enfermedades previas y tratamientos actuales. Para este estudio se recogieron edad materna en años, edad gestacional en días, paridad, cesáreas previas, abortos previos.

Conforme al protocolo asistencial del hospital, todas las mujeres participantes tomaron suplementos de hierro desde la semana 16 de gestación, excepto en caso de enfermedades que lo contraindiquen, como medida preventiva para reducir el riesgo de anemia gestacional y optimizar las reservas de hierro para minimizar el impacto de la pérdida sanguínea en el parto y prevenir la anemia ferropénica en el puerperio, independientemente de los niveles basales de hemoglobina en el postparto.

Las mujeres fueron contactadas en la sala de urgencias y de dilatación, ofreciéndoles la posibilidad de participar en el estudio. La elección de grupo (donante o no donante de sangre de cordón umbilical) fue voluntaria y todas las mujeres firmaron el consentimiento informado para participar en este estudio.

El tipo de pinzamiento del cordón umbilical (precoz: realizado antes del primer minuto de vida del neonato, o tardío: realizado después de un minuto, independientemente de que siga latiendo el cordón o no) quedó a criterio del profesional responsable de la atención al parto.

A la salida del hombro anterior o dentro del primer minuto de vida del neonato se administró a la madre 5 UI de oxitocina por vía intravenosa, según el protocolo del servicio. Se practicó tracción controlada del cordón umbilical cada cinco minutos o cuando aparecieron signos de desprendimiento placentario.

La placenta se expulsó mediante la maniobra de Brandt-Andrews con pujo materno. A continuación se administraron 20 UI de oxitocina vía intravenosa en perfusión continua (500 mL de glucosa al 5% o solución salina al 0,9%).

Se consideró retención placentaria si la placenta no se había expulsado a los 30 minutos. Se efectuaron mediciones temporales desde el nacimiento hasta el final de la sutura perineal, estableciendo puntos intermedios con un cronómetro estándar de *smartphone*.

*Extracción de sangre de cordón umbilical.* El protocolo de extracción, manipulación, almacenamiento y transporte de sangre del cordón umbilical fue proporcionado por el banco público de sangre y tejidos de la Comunidad Valenciana^21^. Si la mujer había aceptado donar la sangre del cordón umbilical, el procedimiento se llevó a cabo en campo estéril inmediatamente después del pinzamiento del cordón umbilical y antes del alumbramiento placentario. La recogida se realizó mediante punción de la vena umbilical con una aguja estéril conectada a una bolsa con anticoagulante, siguiendo las recomendaciones estándar para la recolección de progenitores hematopoyéticos^22^. La extracción fue realizada por personal sanitario capacitado y la sangre recolectada fue transferida para su procesamiento y almacenamiento al biobanco del centro de transfusiones de la Comunidad Valenciana, de acceso y uso público.

*Variables de resultado maternas*

Se consideró existencia de *hemorragia postparto precoz* cuando en el postparto (12-24 horas), y respecto a la medición preparto, se observó una disminución de hemoglobina de 4 g/dL y/o de 10 puntos en el hematocrito, o hubo que transfundir en ese periodo^5^^,^^8^.

Para valorar la pérdida sanguínea se recogieron las variables hemodinámicas hemoglobina (rango normal: >11 g/dL), hematocrito (rango normal: >34%) y presión arterial sistólica y diastólica (PAS y PAD) en mm Hg (cifras normales pregestacionales, <140/90 mm Hg)^23^, tanto antes como después del parto. Con ellas se calculó la diferencia de hemoglobina, la diferencia de hematocrito y la diferencia de presión arterial sistólica y diastólica.

Otras variables estudiadas fueron: duración del alumbramiento en minutos (desde la salida del neonato a la expulsión placentaria), tiempo en minutos transcurrido entre el nacimiento y el final de la sutura, necesidad de transfusión sanguínea, retención placentaria y el tipo de extracción de la placenta.

No se propuso excluir los partos inducidos hasta observar su comportamiento respecto a las variables a estudio.

*Variables de resultado neonatales*

Se recogieron la edad gestacional y el peso del recién nacido, el pH de la arteria y vena umbilicales y el test de Apgar a los cinco y a los diez minutos del nacimiento.

La extracción de sangre para el análisis del pH fue realizada por las matronas que atendían el parto, utilizando jeringas heparinizadas; las medidas de pH se realizaron en el gasómetro de la sala de partos entre uno y dos minutos tras el nacimiento, lo que minimiza la alteración de los resultados por retrasos en el análisis, aumentando la fiabilidad de los valores obtenidos. Se consideró normal un pH >7,15 en sangre arterial y/o venosa umbilical al nacimiento, de acuerdo al criterio de ingreso en la unidad de observación neonatológica del hospital.

El test de Apgar consiste en valorar la adaptación y vitalidad del neonato tras el nacimiento, evaluando cinco parámetros (color de la piel, irritabilidad refleja, tono muscular, frecuencia cardiaca y esfuerzo respiratorio) al primer minuto, a los cinco y a los diez. Se consideró normal un valor de Apgar >7, de acuerdo al criterio de ingreso en la unidad de observación neonatológica del hospital.

Los valores de pH umbilical y de Apgar a un minuto se consideraron parámetros no directamente relacionados con la DSCU, sino dependientes de factores como el momento del pinzamiento del cordón umbilical y el estado neonatal al nacimiento, y no serán usados en la comparación entre grupos.

### Análisis estadístico

Las variables cuantitativas se describieron con la media y la desviación estándar (DE). Para la comparación entre dos grupos, se utilizó el test t de Student cuando se cumplían los supuestos de normalidad y homogeneidad de varianzas, o U de Mann-Whitney en caso contrario. Para comparaciones múltiples se utilizó el test de ANOVA seguido de un *post hoc* en caso de normalidad, o Kruskall-Wallis y de la corrección de Bonferroni en caso contrario. La relación entre variables se estudió mediante la correlación de Pearson (coeficiente r) o de Spearman (coeficiente rho), según las variables tuvieran una distribución normal o no. Las variables cualitativas se describieron con frecuencias y porcentajes y se compararon mediante la prueba de Chi-cuadrado, aplicando el test exacto de Fisher o la corrección de continuidad de Yates con frecuencias esperadas bajas; además, se calculó el riesgo relativo (RR).

Mediante regresión lineal se analizó la relación de las variables relacionadas con la hemorragia postparto precoz con todas las variables maternas, tanto previas al parto (edad materna, gestaciones previas, antecedentes de cesárea y aborto, días de gestación, PAS, PAD, hematocrito y hemoglobina) como propias del parto (tiempo de alumbramiento, tiempo entre el nacimiento y el final de la sutura). Se realizaron dos modelos predictivos, uno para la diferencia de hemoglobina y otro para la diferencia de hematocrito. Las variables se incluyeron mediante un procedimiento por pasos hacia adelante; el modelo final incluyó todas las variables significativas. Se consideraron modelos clínicamente relevantes aquellos que obtenían una R^2^ ajustada >0,250 (Cohen)^24^.

Se consideró un valor de p <0,05 en contraste bilateral como estadísticamente significativo. El análisis se realizó con el programa estadístico SPSS versión 21.

## RESULTADOS

**Figura 1:**
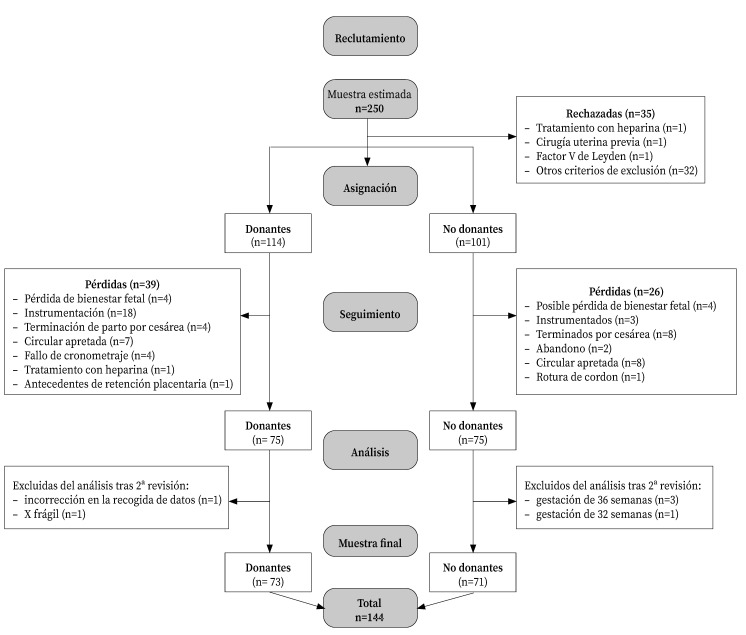
Diagrama de flujo de reclutamiento y seguimiento.

Se obtuvo una muestra final de 144 mujeres (73 donantes y 71 no donantes) que cumplieron con los criterios de inclusión después de las pérdidas (Fig. 1). Para cálculos hemáticos se contabilizaron 12 pérdidas, seis por grupo, por no desear extracción de hematocrito y hemoglobina en las 12-24 horas tras el parto.

Las características demográficas y clínicas basales de las mujeres y sus hijos, incluyendo los valores de Apgar al minuto y el pH umbilical se muestran en la Tabla 1. Las mujeres sin gestaciones previas optaron por la DSCU con mayor frecuencia (63%), mientras que solo el 40% de aquellas con más de dos gestaciones previas optaron por ella. La PAD preparto, fue menor en las donantes.

Ningún pH de vena o arteria umbilical fue menor a 7,12. El 87,64% de los neonatos del grupo de donantes y el 95,78% del grupo de no donantes puntuaron entre 9-10 en el primer minuto en el test de Apgar.

**Tabla 1:** Datos descriptivos de madres y neonatos según donación de sangre del cordón umbilical

	DSCU	No DSCU	p
	n=73	n=71	
*Variables de la madre*			
Basales			
Edad, *media (DE)*	31,48 (5,60)	32,32 (5,04)	0,37^u^
Paridad, *media (DE)*	0,75 (1,01)	0,77 (0,81)	0,56^u^
Gestaciones previas, *n (%)*			0,04^χ²^
0	29 (63,0)	17 (37,0)	
≥1	44 (44,9)	54 (55,1)	
Primíparas, *n (%)*	32 (43,8)	30 (42,7)	0,98^χ²^
Abortos previos, *n (%)*	18 (24,7)	25 (35,2)	0,63^χ²^
Ectópicos previos, *n (%)*	2 (1,4)	1 (0,6)	1,00^f^
Cesáreas previas, *n (%)*	1 (1,4)	7 (9,9)	0,03^f^
Transfusiones previas, *n (%)*	0	1 (0,7)	0,49^f^
*Preparto*			
Hematocrito (%), *media (DE)*	37,12 (3,06)	37,57 (2,56)	0,42^t^
Hemoglobina (gr/dL), *media (DE)*	12,28 (1,04)	12,50 (0,96)	0,24^t^
PAS (mm Hg), *media (DE)*	119,64 (11,13)	121,15 (14,00)	0,51^t^
PAD (mm Hg), *media (DE)*	68,90 (9,56)	72,38 (10,86)	0,06^t^
*Parto*			
Inducción del parto, *n (%)*	29 (39,7)	32 (45,1)	0,41^χ²^
Ammiorrexis, *n (%)*	35 (47,9)	36 (50,7)	0,62^χ²^
Horas de bolsa rota, *media (DE)*	443,04 (447,06)	574,3 (498,90)	0,10^u^
Pinzamiento, *n (%)*			0,95^χ²^
Precoz	50 (50,5)	49 (49,5)	
Tardío	23 (50,0)	23 (50,0)	
*Variables del neonato*			
Edad gestacional (semanas), *media (DE)*	39+5 (6,89)	39+5 (7,38)	0,80 ^t^
Peso (gramos), *media (DE)*	3.317,6 (357,11)	3.293,3 (404,45)	0,60^t^
Apgar a 1 minuto, *media (DE)*	9,37 (0,87)	9,41 (0,62)	0,75^u^
pH arterial, *media (DE)*	7,28 (0,07)	7,29 (0,06)	0,36^u^
pH venoso, *media (DE)*	7,34 (0,05)	7,35 (0,05)	0,23^u^

DSCU: donación de sangre del cordón umbilical; DE: desviación estándar; PAS: presión arterial sistólica; PAD: presión arterial diastólica. Las variables cuantitativas se compararon con t de Student (t) excepto las comparadas con U de Mann-Whitney (u); las variables categóricas se compararon con χ² y con el test exacto de Fisher (f) en caso de bajas frecuencias esperadas.

En la Tabla 2 se pueden observar que ambos grupos fueron comparables respecto al peso medio de los recién nacidos y a las diferencias de hematocrito y hemoglobina pre y postparto. Ninguna mujer con cesárea previa en nuestra muestra presentó hemorragia postparto ni complicaciones hemodinámicas. En las mujeres con cesárea previa realizada al menos dos años antes, el descenso promedio de hematocrito fue de 7,05 puntos y el descenso promedio de hemoglobina de 2,51 g/dL.

Cuantificamos seis hemorragias postparto en el grupo de donantes y una en el de no donantes (p=0,60); la necesidad de transfusión sanguínea no aumentó significativamente por el hecho de realizar DSCU (p=0.30). Una mujer del grupo No DSCU y dos mujeres del grupo DSCU fueron transfundidas, estas dos últimas debido a retención placentaria con extracción manual de la placenta con atonía uterina asociada. Solo aparecieron repercusiones hemodinámicas en las mujeres con hemorragias que recibieron trasfusión.

La duración media del alumbramiento placentario fue superior en el grupo DSCU, sin alcanzar significación estadística. La fase de expulsión fetal fue similar en ambos grupos, y el tiempo medio transcurrido desde el inicio del parto hasta el final de la sutura fue significativamente mayor en las donantes. El tipo de parto fue eutócico en ambos grupos, sin partos instrumentados. No se registraron diferencias en la necesidad de revisión del canal blando ni en la incidencia de desgarros perineales de tercer o cuarto grado entre los grupos. El grupo DSCU presentó mayor proporción de episiotomías, desgarros perineales y casos de retención placentaria, pero sin alcanzar significación estadística. El peso placentario medio fue menor en las donantes. No se encontró relación entre la episiotomía y las diferencias de hemoglobina y hematocrito pre y postparto.

**Tabla 2:** Variables de resultado de madres y neonatos según donación de sangre del cordón umbilical

	DSCU	No DSCU	p
	n=73	n=71	
*Variables de la madre*			
Analgesia raquídea, *n (%)*	48 (65,8)	52 (73,2)	0,33^χ²^
Episiotomía, *n (%)*	39 (46,6)	28 (39,4)	0,09^χ²^
Desgarro 3er grado, *n (%)*	2 (2,7)	0	0,5
Retención placentaria, *n (%)*	4 (5,5)	1 (1,41)	0,36^f^
Extracción manuales de la placenta, *n (%)*	2 (2,7)	0	0,50^f^
Duración (minutos), *media (DE)*			
Dilatación	229,05 (228,43)	266,83 (275,66)	0,37^u^
Expulsivo	44,77 (43,60)	50,38 (46,14)	0,45^u^
Tiempo (minutos), *media (DE)*			
Al alumbramiento	11,11 (10,05)	7,99 (5,92)	0,10^u^
Nacimiento-final de sutura	30,69 (16,37)	22,91 (12,19)	<0,001^t^
Diferencia preparto-postparto, *media (DE)*			
Hematocrito	3,63 (4,25)	3,23 (3,07)	0,53^t^
Hemoglobina	1,31 (1,42)	1,20 (1,02)	0,63^u^
TAD	2,00 (14,7)	3,81 (12,09)	0,43^u^
TAS	2,06 (14,66)	2,68 (12,22)	0,78^u^
Necesidad de transfusión, *n (%)*	2 (2,70)	0	0,50^f^
Hemorragia postparto, *n (%)*	6 (0,83)	1 (0,14)	0,17^f^
*Variables del neonato*			
Peso (gramos), *media (DE)*	3.317,60 (357,11)	3.293,31 (404,45)	0,60^t^
Peso placentario (gramos), *media (DE)*	558,36 (92,81)	599,72 (132,92)	0,03^u^
Apgar, *media (DE)*			
5 minutos	9,93 (0,30)	10 (0,00)	0,06^u^
10 minutos	9,96 (0,20)	10 (0,00)	0,08^u^

DSCU: donación de sangre del cordón umbilical; DE: desviación estándar; PAS: presión arterial sistólica; PAD: presión arterial diastólica. t: t de Student; u: U de Mann-Whitney; χ2: Chi cuadrado; f: test exacto de Fisher.

Los valores del test de Apgar neonatal no mostraron diferencias significativas (U de Mann-Whitney) en relación al pinzamiento precoz o tardío del cordón umbilical, ni a los cinco minutos (9,37; DE=0,75 frente a 9,43; DE=0,79; p=0,65) ni a los diez minutos (9,96; DE=0,24 frente a 9,98; DE=0,15; p=0,60).

La DSCU no aumentó significativamente el RR de necesidad de transfusión sanguínea (n=2 frente a n=0; RR=4,92; IC95%: 0,25-98,76), retención placentaria (n=4 frente a n=1; RR=3,89; IC95%: 0,45-33,97) ni extracción manual de placenta (n=2 frente a n=0; RR=4,92; IC95%: 0,25-98,76) frente al grupo no donante. Tampoco el parto inducido aumentó el RR de hemorragia postparto (RR=2,9; IC95%: 0,31-27,27) ni de hemorragia postparto precoz (RR=4,8; IC95%: 0,24-99,19).

Se encontraron correlaciones moderadas y positivas (p<0,001) entre el tiempo de alumbramiento placentario y la diferencia pre y postparto de hematocrito (r=0,317) y de hemoglobina (r=0,301), y entre el tiempo transcurrido desde el nacimiento hasta el final de la sutura y las diferencias de hematocrito (r=0,337) y de hemoglobina (r=0,315). Se encontraron correlaciones débiles y negativas (p<0,05) entre la duración del periodo de dilatación y el pH venoso fetal (r=-0,186) y el Apgar a los 5 minutos (r=-0,190), y entre el tiempo desde el nacimiento al final de la sutura con el pH arterial (r=-0,173). Un mayor peso fetal se correlacionó (p<0,05) con una mayor disminución de la hemoglobina (r=0,185), una mayor diferencia de TAS (r=0,169) y un mayor número de desgarros perineales (0,169).

La PAS preparto, la realización de episiotomía, la ocurrencia de desgarro perineal y la extracción manual de la placenta, aumentaron la diferencia de hematocrito y hemoglobina (valores preparto frente a valores postparto), mientras que el antecedente de aborto la disminuyó (Tabla 3).

También se analizaron estas diferencias de hematocrito y hemoglobina desagregadas según las gestaciones. Aunque los modelos no fueron clínicamente relevantes, las variables predictoras de la diferencia, tanto de hematocrito como de hemoglobina, fueron el tipo de alumbramiento en primigestas, la presencia de cesárea previa en mujeres con dos gestaciones, y el hematocrito previo al parto en aquellas con más de dos gestaciones.

**Tabla 3:** Modelos de regresión lineal múltiple predictivos para la diferencia de hematocrito y de hemoglobina

	Diferencia de hematocrito		Diferencia de hemoglobina	
R^2^ ajustada	0,312		0,343	
Variables	B	p	B	p
Constante	-10,233	0,002	-3.937	<0,001
Abortos	-1,21	0,008	-0,446	0,003
Episiotomía	2,914	0,001	1,052	0
PAS preparto	0,059	0,012	0,025	0,001
Desgarro perineal	1,391	0,006	0,512	0,002
Extracción manual de la placenta	4,525	0,001	1,527	0,001
Tiempo nacimiento-final de sutura (minutos)	0,005	0,86	-0,002	0,854

## DISCUSIÓN

En este estudio, la DSCU no se asoció con un deterioro de las variables hemodinámicas postparto (hemoglobina, hematocrito, PAS, PAD) ni con el Apgar del neonato a los cinco y diez minutos. Sí se observó un aumento no significativo del tiempo de alumbramiento en las donantes (3,12 minutos), diferencia que concuerda con estudios previos que atribuyen un aumento del tiempo de procedimiento de 3 a 5 minutos a consecuencia de la DSCU^16^.

La DSCU se realiza inmediatamente después del nacimiento, mediante la punción de la vena umbilical. El posparto inmediato es el periodo de mayor morbimortalidad materna, siendo la hemorragia postparto una de las complicaciones más peligrosas^5^^-^^7^. El volumen intraplacentario después del nacimiento puede influir en la duración del alumbramiento placentario, y la DSCU representa una disminución de ese volumen. Es importante destacar que la DSCU está en estrecha relación con el momento del pinzamiento del cordón umbilical, ya que para la recolección de sangre del cordón es necesario pinzar el cordón en el primer minuto de vida, lo que puede tener implicaciones en la transfusión placentaria al neonato. En este estudio, el momento del pinzamiento quedó a criterio del profesional, lo que representa un factor a controlar en futuros estudios. Aunque en el periodo periparto se pueden evaluar múltiples variables, algunas potenciales consecuencias del pinzamiento precoz y de la DSCU, como la repercusión en los depósitos férricos neonatales, requieren un seguimiento a medio y largo plazo.

Los estudios publicados presentan una gran heterogeneidad metodológica en el uso de oxitócicos, tracción controlada del cordón umbilical, maniobras de extracción placentaria y realización de drenaje o donación de sangre del cordón umbilical. La primera revisión Cochrane del 2008^13^ apreciaba dudas por la escasez y variabilidad de los diseños y solo revisaba estudios sobre drenaje. Los estudios con DSCU realizados han sido escasos, retrospectivos y sin medición del tiempo de alumbramiento^16^^,^^25^^,^^26^.

El tiempo de alumbramiento no presentó diferencias significativas entre donantes y no donantes, coincidiendo con estudios previos^15^^,^^27^. Sin embargo, existen investigaciones con drenaje de sangre de cordón umbilical que sí observan una reducción significativa del tiempo de alumbramiento^14^^,^^28^^-^^34^. El mayor tiempo de expulsión placentaria observado en las donantes respecto de las mujeres que realizaron el drenaje de sangre del cordón umbilical, podría estar relacionado con el tiempo adicional requerido para vaciar la placenta mediante el procedimiento de donación en sí^16^.

En este estudio se ha observado una disminución de peso placentario en el grupo de donantes, debida a que después de la donación las placentas de las mujeres donantes están exangües; este hallazgo no se podido contrastar con estudios previos.

La pérdida sanguínea (disminución significativa de hematocrito y hemoglobina) y la necesidad de transfusión se emplearon para evaluar la hemorragia posparto, sin observar diferencias significativas entre donantes y no donantes. A pesar de que nuestro estudio se limitó a partos eutócicos o normales, los hallazgos son consistentes con los descritos en el estudio de Rashmi y col^35^, que también incluyó partos instrumentales. La baja frecuencia de necesidad de transfusión sanguínea no permitió calcular con fiabilidad el riesgo relativo de donar. Es importante señalar que las dos transfusiones en el grupo DSCU fueron debidas a casos de retención placentaria con extracción manual de la placenta y atonía uterina, factores bien documentados como causas de hemorragia posparto^8^. La disminución del volumen intraplacentario debido al drenaje ejerció un efecto protector frente a la hemorragia postparto precoz en algunos estudios^16^^,^^28^, mientras otro no observó distinta frecuencia de hemorragia posparto entre grupos de donantes y no donantes^36^.

En nuestro estudio, la donación no se asoció a un aumento de retenciones placentarias ni de extracciones manuales de placenta, coincidiendo con otros autores que tampoco observan modificación por el hecho de drenar, donar o no^15^^,^^16^^,^^28^ o en relación a las extracciones manuales de placenta^16^^,^^26^^,^^28^^,^^37^. En estudios recientes se ha observado una disminución significativa de las retenciones placentarias tras practicar drenaje^30^^,^^31^^,^^34^.

El aumento del tiempo de alumbramiento se correlacionó con una disminución del hematocrito, de la hemoglobina y del pH venoso fetal, ampliamente citado en la literatura^8^^,^^38^^,^^39^. Se podría postular que la aplicación de oxitócicos preventivos, junto con la DSCU, produce una contracción uterina homogénea en toda su extensión y, conforme se va desprendiendo la placenta, el útero se va adaptando a la misma. Sin oxitócicos y en donantes, probablemente la contracción uterina no sea tan rápida y tampoco el tono en la pared muscular sea homogéneo, formándose así un coágulo intracavitario mayor, que aumentaría la presión hacia el cuello uterino, lo que a su vez produciría una adaptación uterina al nuevo volumen (no tan rápida como la oxitócica) que podría disminuir el tiempo de expulsión placentaria. El peso del coágulo formado al haber menor sangrado sería menor en los estudios con oxitocina profiláctica^11^. Ese acortamiento del tiempo de expulsión placentaria, aunque mínimo, podría enmascarar el efecto de la DSCU sobre el tiempo de alumbramiento y la tendencia a la disminución de los niveles de hematocrito y hemoglobina, aunque sin alcanzar significación estadística, que aparece en las donantes.

La correlación positiva entre la duración del periodo de dilatación y el Apgar a los 5 minutos podría deberse al estado hipóxico producido por la prolongación en el tiempo de las contracciones uterinas, dando lugar a una disminución del flujo de paso efectivo acumulado en la vena umbilical.

La correlación entre el tiempo transcurrido desde el nacimiento al final de la sutura y la diferencia pre y postparto de hemoglobina y hematocrito es congruente ya que el avance en el tiempo de los procedimientos de reparación vagino-perineales suele ir ligado a un aumento del sangrado, con la consiguiente disminución de hematocrito y hemoglobina. Asimismo, el aumento del tiempo transcurrido desde el nacimiento al alumbramiento placentario conlleva un aumento de la diferencia pre y postparto de hemoglobina y hematocrito^9^. El tiempo trascurrido desde el parto al final de la sutura fue superior en las donantes y se correlacionó con una disminución del pH arterial umbilical, probablemente debido a la técnica de donación^16^. No obstante, este incremento temporal no se tradujo en diferencias estadísticamente significativas entre grupos en el Apgar a los 5 y 10 minutos.

El descenso promedio observado de hemoglobina (2,51 g/dL) y hematocrito (7,05%) en mujeres con cesárea previa realizada con más de dos años de anterioridad, representan cifras normales en el contexto del parto vaginal^40^.

El peso fetal se ha correlacionado en multitud de publicaciones con la disminución de hemoglobina postparto, el aumento en la diferencia de PAS y la aparición de desgarro perineal; a mayor peso fetal, mayor probabilidad de desgarro perineal^41^.

Este estudio presenta varias limitaciones. La donación de sangre de cordón umbilical es un acto altruista que eliminó toda posibilidad de aleatorización, limitando el diseño del estudio. El hospital experimentó un cambio de ubicación que afectó al número de donaciones y a la dinámica del estudio, lo que provocó una recogida mínima o nula de muestras en ciertos periodos. Las bajas frecuencias observadas de algunas variables dependientes (como necesidad de transfusión sanguínea, retención placentaria o extracción manual de placenta) no permiten comparar con fiabilidad los grupos ni calcular el RR de donar frente a no donar, debido a la falta de potencia estadística, por lo que los resultados deben interpretarse con cautela. El bajo número de mujeres con cesárea previa puede afectar a la representatividad de la muestra. No se incluyó la variable “volumen de sangre donada” ya que en el centro de recogida solo se consignó si el volumen de la muestra feu adecuado o no. La antigüedad del estudio es otra limitación, ya que se han producido cambios en el procedimiento del pinzamiento del cordón umbilical. Actualmente el inicio del pinzamiento se realiza a partir de un minuto desde el nacimiento. Asimismo, aunque el estudio analizó la hemorragia postparto precoz, la necesidad de transfusión y otras variables relacionadas, la incidencia mínima de la misma sugiere interpretar los resultados con cautela.

Consideramos una fortaleza del estudio la tracción controlada de cordón umbilical de forma intermitente (cada cinco minutos) como parte del manejo activo del alumbramiento, ya que minimizó el sesgo que hubiera provocado una tracción continua; este hecho no ha sido contemplado en ningún estudio anterior. La recogida de muestras sin limitación de tiempo en el pinzamiento, no recomendable en la actualidad, ha permitido el análisis de esta variable (pinzamiento temprano/tardío).

En conclusión, y según las condiciones de este estudio, la donación de sangre del cordón umbilical no aumenta la incidencia de hemorragia posparto precoz ni afecta negativamente el estado del neonato al nacimiento en términos de Apgar a cinco y diez minutos, por lo que sería un método seguro tanto para la madre como para el neonato.

El estudio de la hemorragia postparto en partos instrumentados y en cesáreas, y considerar otras variables de la madre y del niño, como el tipo de pinzamiento, y otros factores influyentes en la duración del tiempo desde el parto al final de la sutura, así como la evolución de las reservas de hierro en el neonato a medio y a largo plazo, constituyen aspectos relevantes en la evolución del postparto que podrían ser consideradas en futuras investigaciones para un análisis más completo del fenómeno estudiado.

## Data Availability

Se encuentran disponibles bajo petición al autor de correspondencia.

## References

[1] Chao NJ Collection and storage of umbilical cord blood for hematopoietic cell transplantation.

[2] Fundación Josep Carreras contra la leucemia REDMO 2023.

[3] Hernandez D, Danby RD, Querol S (2022). Umbilical cord blood and tissue in novel therapies and haematopoiesis research. Front Cell Dev Biol.

[4] Organización Nacional de Trasplantes Plan nacional de sangre de cordón umbilical 2020-2025.

[5] World Health Organization (2009). WHO Guidelines for the management of postpartum haemorrhage and retained placenta.

[6] World Health Organization (2012). WHO recommendations for the prevention of postpartum haemorrhage.

[7] Bienstock JL, Eke AC, Hueppchen NA (2021). Postpartum hemorrhage. N Engl J Med.

[8] Guasch E, Gilsanz F (2016). Hemorragia masiva obstétrica: Enfoque terapéutico actual. Med Intensiva.

[9] Angarita AM, Cochrane E, Bianco A, Berghella V (2023). Prevention of postpartum hemorrhage in vaginal deliveries. Eur J Obstet Gynecol Reprod Biol.

[10] Whittington JR, Ghahremani T, Whitham M, Phillips AM, Spracher BN, Magann EF (2023). Alternate birth strategies. Int J Womens Health.

[11] Gallos ID, Williams HM, Price MJ, Merriel A, Gee H, Lissauer D (2018). Uterotonic agents for preventing postpartum haemorrhage: A network meta-analysis. Cochrane Database Syst Rev.

[12] Díaz-Rossello JL (2006). Early umbilical cord clamping and cord-blood banking. The Lancet.

[13] Soltani H, Dickinson F, Leung TN (2005). The effect of placental cord drainage in the third stage of labour on feto-maternal transfusion: A systematic review. Evid-Based Midwifery.

[14] Wu HL, Chen XW, Wang P, Wang QM (2017). Effects of placental cord drainage in the third stage of labour: A meta-analysis. Sci Rep.

[15] Vasconcelos FB, Katz L, Coutinho I, Lins VL, de Amorim MM (2018). Placental cord drainage in the third stage of labor: Randomized clinical trial. PLoS One.

[16] Guillaume A, Sananès N, Poirier V, Gaudineau A, Fritz G, Boudier E (2015). Benefits of cord blood collection in the prevention of post-partum hemorrhage: a cohort study. J Matern Fetal Neonatal Med.

[17] Ogba EI, Chukwudi NK, Izuka OM, Adizua UC (2024). Prevalence of perinatal asphyxia using apgar scores and cord blood ph and the relationship between the two methods: A study of FMC Umuahia. Niger J Clin Pract.

[18] Dirección General para la Salud Pública. Servicio de Salud Infantil y de la Mujer. Generalitat Valenciana. Conselleria de Sanitat (2002). https://biblioesp.gva.es/publicos/tsan/documentos/mig/V.1516-2002.pdf.

[19] Centro de Transfusiones de la Comunidad Valenciana Cuestionario de evaluación donantes de Sangre de Cordón Umbilical.

[20] Sánchez Ortiz M, López Pérez M, Sánchez Muñoz A, Gil Raga F, Aguilar Galán V (2019). Incidencia y factores de riesgo en la hemorragia postparto precoz. Apuntes de Ciencia.

[21] Centro de Transfusiones de la Comunidad Valenciana Consentimiento informado donación de Sangre de Cordón Umbilical.

[22] Hidalgo M, Molina L (2013). Procedimiento para la donación de sangre del cordón umbilical. Inquietudes.

[23] García-Penche Santillán E, López López C, Boguñá Ponsa JM, Laílla Vicens JM, González Bosquet E (2024). Gonzalez Merlo Obstetricia.

[24] Cohen J (2013). Statistical power analysis for the behavioral sciences.

[25] Amat L, Sabrià J, Martínez E., Rodríguez NL, Querol S, Lailla J (2011). Cord blood collection for banking and the risk of maternal hemorrhage. Acta Obstet Gynecol Scand.

[26] Mlodawski J, Mlodawska M, Przybysz N, Bielak A, Detka K, Pasiarski M (2021). Collection of umbilical cord blood and the risk of complications in postpartum women after natural labour in the context of the possibility of umbilical cord stem cells usage in clinical practice. Ginekol Pol.

[27] Javeed M, Parveen S, Yaseen A, Bajwa Z, Nawaz N, Azhar M (2022). Effect of placental cord blood drainage on duration of third stage of labour. Pak J Med Sci.

[28] Asicioglu O, Unal C, Asicioglu BB, Temizkan O, Yildirım G, Arici B (2015). Influence of placental cord drainage in management of the third stage of labor: a multicenter randomized controlled study. Am J Perinatol.

[29] Chaudhary M, Shah M, Makwana N (2020). Placental cord drainage during third stage of labour: A randomized control trial at a tertiary care centre. Int J Reprod Contracept Obstet Gynecol.

[30] El-Said Mansour S, Hemida R, Mohamed Ibrahim Gouda A (2021). Placental cord drainage: Its effect on duration and blood loss of third stage of labor. Egypt J Health Care.

[31] Karimi N, Molaee G, Tarkesh Esfahani N, Montazeri A (2022). Placental cord drainage and its outcomes at third stage of labor: A randomized controlled trial. BMC Pregnancy Childbirth.

[32] Ghani SA, Janjua M, Mubeen S, Hussain M, Jawad Z (2020). Comparison and the duration of third stage of labour with or without cord blood drainage in females undergoing normal vaginal delivery at term. J Soc Obstet Gynaecol Pak.

[33] Nabil H, Marzouk T (2020). Placental cord drainage versus clamping for prevention of blood loss in the third stage of labour. MJCU.

[34] Dosedla E, Gašparová P, Ballová Z, Sitáš M, Calda P (2023). Effect of umbilical cord drainage after spontaneous delivery to the third stage of labor. Ceska Gynekol.

[35] Rashmi MB, Hadalagi NM (2018). Randomised prospective study of placental blood drainage for the prevention of postpartum hemorrhage. Int J Clin Obstet Gynaecol India.

[36] Hendin N, Grosvald M, Shemesh I, Leonenko M, Jbara M, Segal K (2024). Placental cord drainage impact on third stage of labor: A randomized controlled trial. Am J Obstet Gynecol MFM.

[37] Bostanci E, Kiliççi Ç, Kumru P, Yayla Ç, Darici E, Eroğlu M (2019). Placental drainage versus no placental drainage after vaginal delivery in the management of third stage of labour: A randomized study. Zeynep Kamil Tıp Bülteni.

[38] Oberg AS, Hernandez-Diaz S, Palmsten K, Almqvist C, Bateman BT (2014). Patterns of recurrence of postpartum hemorrhage in a large population-based cohort. Am J Obstet Gynecol.

[39] Aroviita P, Teramo K, Hiilesmaa V, Kekomäki R (2005). Cord blood hematopoietic progenitor cell concentration and infant sex. Transfusion.

[40] Castilla M, Donado C, Hijona JJ, Jaraíz MVE, Santos MJ (2015). ¿Conocemos los factores asociados al descenso de hemoglobina en el posparto?. Clínica e Investigación en Ginecología y Obstetricia.

[41] Wilson AN, Homer CSE (2020). Third- and fourth-degree tears: A review of the current evidence for prevention and management. Aust N Z J Obstet Gynaecol.

